# Editorial: Open-access data, models and resources in neuroscience research

**DOI:** 10.3389/fnins.2023.1142317

**Published:** 2023-02-14

**Authors:** Ivan Zaletel, Richard S. Nowakowski, Torbjørn V. Ness

**Affiliations:** ^1^Institute of Histology and Embryology “Aleksandar Ð. Kostić”, Faculty of Medicine, University of Belgrade, Belgrade, Serbia; ^2^Department of Biomedical Sciences, Florida State University, College of Medicine, Tallahassee, FL, United States; ^3^Faculty of Science and Technology, Norwegian University of Life Sciences, Ås, Norway

**Keywords:** open-access, open data (OD), neuroscience databases, neuroimaging data repository, data sharing, big data, data protection, data mining

## Why open-access data?

Life science has traditionally not been known for the open sharing of data and models. When Hopfield, a pioneer in computational neuroscience, attended his first biology conference in 1970, he spoke of his experience: “One of the senior biochemists took me aside to explain why I had no place in biology. As he said, gentlemen did not interpret other gentlemen's data and preferably worked on different organisms. If you wish to interpret data, you must get your own.” (Hopfield, 2014). The culture has however shifted and making use of open-access data, models and resources has now become commonplace in neuroscience. There are many good reasons for this, including the high quality of the available data, along with increased reproducibility and transparency, and collaboration opportunities among researchers working in different institutions, countries, and continents (Choudhury et al., 2014; Pascu and Burgelman, 2022). The tendency of making use of publicly available data gained further momentum in the past few years when the COVID-19 pandemic led to a near halt in experimental laboratory work in some countries. Thus, remote working and remote analysis of open-access databases emerged as a solution for overcoming the lack of hands-on laboratory work and were recommended by several neuroscience committees and societies (Vlisides et al., 2021).

The most important reason for the increasing use of open-access data, models, and resources is perhaps simply the increased availability. During the previous decade, there has been a tremendous increase in the number of published neuroscience papers accompanied by raw experimental data, spurring the creation of numerous data-sharing initiatives and databases that provide, at no cost, a large number of datasets (Spires-Jones et al., 2016). These databases can be used for various forms of neuroscientific research, such as morphometric analysis of nervous system cells (Ascoli et al., 2007), neuroimaging analysis of MRI datasets (Poldrack and Gorgolewski, 2014; Markiewicz et al., 2021), as well as genomic, transcriptomic, and proteomic expression analysis (Pereira et al., 2014; Keil et al., 2018). Needless to say, multiple users for these databases leverages the value of the original investment both in time and money that was needed to create them.

The same progress has been seen for open-access models as well: In the past, when computational neuroscientists wanted to reproduce modeling results, they often had to re-implement models based on equations and tables of parameters from scientific papers. This obstacle to scientific progress and reproducibility was acknowledged early, and the ModelDB^1^ repository for sharing neuroscience models was founded in 1996 and has been continuously growing ever since (McDougal et al., 2017). More recently, we have also seen the rise of several new model repositories, both from large initiatives such as the Allen Brain Institute^2^ and the Human Brain Project and from community-based efforts, such as the Open Source Brain.^3^

## Research Topic

The main idea behind this Research Topic was to bring to the attention of a wide audience the use of open-access data, models, and resources from different publicly available repositories to generate new insights. Thus, we welcomed research papers based on open-access data or models, as well as any form of new computational resource, software, or platform that could be used for neuroscience research. As a result, eight articles were published in this topic that cover diverse aspects of neuroscience research, ranging from basic to clinical neuroscience, exploration of genetic and MRI databases, and different software tools for handling connectomic, electrophysiological, and multi-modal data. As this Research Topic has shown, the greatest interest in using open-access data is still present in research based on the use of neuroimaging databases, primarily containing MRI images (Short et al.; Beauferris et al.; Saat et al.). This is not surprising as these types of data can be easily obtained from diverse and large populations of human subjects, both healthy and diseased. In this way, researchers are offered a large pool of data that can be analyzed and lead to new advances in diagnosing and managing neuropsychiatric and other disorders (Poline et al., 2012). Another important component of this topic was the papers in which novel software tools and/or algorithms were presented. These include a unified framework for handling *in vivo* high-density extracellular probes (Garcia et al.), large-scale data analysis in connectomic research (Plaza et al.), as well as a query tool that can be used to easily search and categorize published multi-modal papers (Li and Liang). Further, the topic includes original research providing novel insights into the possible genes underlying autism spectrum disorders (Li et al.), which again emphasizes the importance and potential usage of open-access data obtained from gene-expression studies. Although there is no doubt that open-access data will yield discoveries in the field of neuroscience, the question of protection and privacy of such data arises (Jwa and Poldrack, 2022). This remains one of the most important and controversial questions in the world of open-access data sharing, and this topic includes an opinion article on the current problems, obstacles, and policies regarding data sharing (Lathe).

## Perspectives

Although open-access databases have been present in neuroscience research for more than a decade, their importance is growing and is likely to continue to grow in the coming years (Wiener et al., 2016). This may represent a game-changing option for early-career researchers, or researchers in countries with less-than-optimal science funding as it gives access to resources that would otherwise have been restricted to large and well-funded labs. The increasing availability of online computational resources, like the EBRAINS^4^ or Neuroscience Gateway,^5^ can also be expected to contribute to making high-quality scientific work within reach of a much larger population of aspiring scientists. These initiatives are not only helping researchers gain access to large-scale computational resources but furthermore, have commonly used neuroscientific software pre-installed. This makes it almost trivial for researchers to ensure that their simulation results are reproducible and interactable since the computational environment that produced the results can easily be fully specified and recreated.

Open-access data, models, and tools are valuable only as long as they are Findable, Accessible, Interoperable, and Reusable (FAIR) (Wilkinson et al., 2016). Achieving this requires a joint effort: On the one hand, those responsible for the database or repository must facilitate that it is easy to search through and access, both for humans and machines, and that interacting with it does not require the user to be an expert programmer. On the other hand, researchers must strive to be sufficiently proficient in handling the variety of software needed to interact with online databases and repositories. This underscores the importance of making bioinformatics training an essential feature in biomedical curricula, or else many of these databases will remain underutilized and will not reach their full potential (Akil et al., 2011).

Neuroscience is increasingly becoming the highly interconnected global endeavor it needs to be to overcome the challenges ahead. As further progress is made, we look forward to the increasing usage of ever-expanding databases, leading to further advances in neuroscience.

## Author contributions

IZ wrote the first draft of the article. RN and TN revised the final version of the manuscript. All authors read and approved the final submitted version.

## Footnotes

^1^ https://senselab.med.yale.edu/ModelDB/

^2^ https://portal.brain-map.org/

^3^ https://www.opensourcebrain.org/

^4^ https://ebrains.eu/

^5^ https://www.nsgportal.org/
